# A decision support system to recommend appropriate therapy protocol for AML patients

**DOI:** 10.3389/frai.2024.1343447

**Published:** 2024-03-06

**Authors:** Giovanna A. Castro, Jade M. Almeida, João A. Machado-Neto, Tiago A. Almeida

**Affiliations:** ^1^Department of Computer Science, Federal University of São Carlos (UFSCar) Sorocaba, São Paulo, Brazil; ^2^Institute of Biomedical Sciences, The University of São Paulo (USP), São Paulo, Brazil

**Keywords:** Acute Myeloid Leukemia, risk classification, prognostic prediction, supervised learning model, machine learning, decision support system

## Abstract

**Introduction:**

Acute Myeloid Leukemia (AML) is one of the most aggressive hematological neoplasms, emphasizing the critical need for early detection and strategic treatment planning. The association between prompt intervention and enhanced patient survival rates underscores the pivotal role of therapy decisions. To determine the treatment protocol, specialists heavily rely on prognostic predictions that consider the response to treatment and clinical outcomes. The existing risk classification system categorizes patients into favorable, intermediate, and adverse groups, forming the basis for personalized therapeutic choices. However, accurately assessing the intermediate-risk group poses significant challenges, potentially resulting in treatment delays and deterioration of patient conditions.

**Methods:**

This study introduces a decision support system leveraging cutting-edge machine learning techniques to address these issues. The system automatically recommends tailored oncology therapy protocols based on outcome predictions.

**Results:**

The proposed approach achieved a high performance close to 0.9 in F1-Score and AUC. The model generated with gene expression data exhibited superior performance.

**Discussion:**

Our system can effectively support specialists in making well-informed decisions regarding the most suitable and safe therapy for individual patients. The proposed decision support system has the potential to not only streamline treatment initiation but also contribute to prolonged survival and improved quality of life for individuals diagnosed with AML. This marks a significant stride toward optimizing therapeutic interventions and patient outcomes.

## 1 Introduction

Acute Myeloid Leukemia (AML) is a highly aggressive hematological malignancy characterized by the infiltration of cancer cells in the bone marrow. It is associated with lower remission rates as patients age, and the average overall survival rate ranges from 12 to 18 months (Rose-Inman and Kuehl, 2014; Pelcovits and Niroula, 2020).

The European Leukemia Net (ELN) established guidelines for diagnosing and treating AML in 2010 (Döhner et al., 2010). These served as a cornerstone in the field, providing valuable insights. Subsequent updates were published in 2017 (Döhner et al., 2017) and 2022 (Döhner et al., 2022), reflecting advancements in understanding AML's biomarkers, disease subtypes, and overall behavior. These updates have contributed to a more comprehensive and up-to-date disease management approach.

According to the current diagnostic criteria for AML, established by the World Health Organization (WHO), the presence of at least 10 or 20% myeloblasts in the bone marrow or peripheral blood is required, depending on the specific molecular subtype of the disease (Arber et al., 2022). These guidelines, outlined in the Classification of Tumours of Haematopoietic and Lymphoid Tissues, provide standardized criteria for diagnosing AML accurately.

Apart from the initial diagnosis, patients with AML also undergo a prognostic evaluation to determine their risk profile, typically categorized into favorable, intermediate, and adverse. This risk stratification relies on analyzing cytogenetic and molecular characteristics (The Cancer Genome Atlas Research Network, 2013). Cytogenetic characteristics involve specific chromosome alterations, while mutations in genes such as *NPM1, RUNX1, ASXL1, TP53, BCOR, EZH2, SF3B1, SRSF2, STAG2*, and *ZRSR2* determine molecular characteristics. Healthcare professionals extensively employ the ELN risk classification to make critical treatment decisions, as it directly influences the patient's prognosis, quality of life, and overall survival.

The main problem with the current ELN risk classification is the significant variability within the same risk group. Accurately assessing the intermediate-risk group is especially challenging, potentially causing delays in starting treatment and worsening patients' conditions. To address this problem, we present a decision support system that automatically recommends therapeutic protocols for AML patients based on their survival prediction. By minimizing subjectivity and streamlining the decision-making process, the proposed approach can enhance patient outcomes, extending survival time and improving overall quality of life.

## 2 Related work

Treatment decisions for AML rely heavily on predicting the patients' response and clinical outcomes, primarily based on cytogenetic factors (Estey, 2019). However, significant heterogeneity within the same risk groups results in diverse outcomes, ranging from rapid decease to unexpected remission (Döhner et al., 2010).

Chemotherapy has been the established standard therapy since the mid-1970's, but its effectiveness in terms of survival rates has been limited (Bennett et al., 1976). Recent advancements have facilitated the collection and analysis of extensive data on genetic mutations and gene expressions (The Cancer Genome Atlas Research Network, 2013), leading to novel therapeutic strategies and a more targeted approach to treatment. These have opened up new possibilities for improving the outcomes and overall management of AML patients.

In 1976, a study conducted by an international collaboration of French, American, and British researchers known as the FAB (French-American-British) group introduced a classification system for AML. Based on the analysis of morphological characteristics in the bone marrow and peripheral blood, this classification system aimed to stratify AML patients into distinct subtypes. The FAB classification scheme defined six subtypes (M1, M2, M3, M4, M5, and M6) based on the differentiation and maturation levels of the leukemic cells. This classification system was a fundamental framework for understanding and characterizing AML, contributing to subsequent research and guiding clinical approaches (Bennett et al., 1976).

In 2010, the European Leukemia Net (ELN) introduced a novel risk classification system that considers cytogenetic and molecular information, providing a more comprehensive assessment of disease severity (Döhner et al., 2010). This updated scheme includes four risk categories: favorable, intermediate I, intermediate II, and adverse. While this stratification system offers improved accuracy compared to traditional cytogenetic analysis, it is challenging to implement it during the initial clinical evaluation due to the high costs associated with sample collection and the subsequent molecular analyses required. Nonetheless, this risk classification plays a crucial role in guiding treatment decisions and optimizing patient outcomes in the management of AML.

A significant update to the ELN's guidelines was published in 2017 based on findings regarding AML behavior (Döhner et al., 2017). The updated risk classification grouped patients into three categories (favorable, intermediate, and adverse) and refined the prognostic value of specific genetic mutations. Since then, specialists have commonly used this stratification to support important decisions about the course of each treatment, which can directly impact the patient's quality of life and life expectancy.

In 2022, the ELN updated its risk classification system, incorporating significant changes based on emerging research findings. One notable revision includes the *FLT3-ITD* gene expression as a key determinant. Patients with high expression levels of this gene, lacking other adverse risk characteristics, are now categorized as intermediate risk. Furthermore, mutations in genes such as *BCOR, EZH2, SF3B1, SRSF2, STAG2*, and *ZRSR2* are now associated with the adverse risk classification (Döhner et al., 2022). These updates reflect new insights regarding the impact of these genetic factors on disease progression and treatment outcomes (The Cancer Genome Atlas Research Network, 2013; Angenendt et al., 2019). The evolving understanding of these molecular characteristics provides valuable information for risk stratification and personalized management of AML patients.

Patients with a favorable risk profile typically exhibit favorable responses to chemotherapy. Conversely, those with an adverse risk profile often display limited responsiveness to standard chemotherapy and may require alternative treatments, such as Hematopoietic stem cell transplantation (The Cancer Genome Atlas Research Network, 2013). However, the therapeutic response of AML patients with an intermediate risk profile remains less clearly defined. The heterogeneous nature of this subgroup makes it challenging to predict their specific treatment outcomes, demanding further research and tailored approaches to optimize their clinical management.

The current risk classifications present challenges due to significant variability within the same risk group. Factors such as age and gender can significantly influence treatment outcomes. For instance, patients under 60 years old tend to respond better to high-dose chemotherapy. In comparison, patients over 60 years old may have a lower tolerance for intense chemotherapy and require alternative palliative therapies (Lagunas-Rangel et al., 2017). However, current risk classifications do not consider age a relevant factor in treatment decision-making. As a result, even among patients classified as having intermediate risk, specialists often rely on additional information, such as results from other tests and analyses, to determine the most appropriate therapy despite limited evidence of efficacy (Döhner et al., 2010). This reliance on supplementary information can delay treatment initiation and worsen the patient's clinical condition. Therefore, there is a need for improved risk stratification models that consider diverse patient characteristics to ensure more precise and timely therapy decisions in AML.

To address these challenges, recent studies have applied machine learning (ML) techniques to predict patient survival and treatment outcomes. By leveraging ML algorithms, researchers aim to automate the prediction of patient response to specific treatments and the likelihood of achieving complete remission. These ML models can handle huge clinical and molecular features to compute predictions, allowing for a more data-driven approach to treatment decision-making. The main goal is to provide valuable insights and assist clinicians in making informed decisions that can optimize patient outcomes and improve the overall management of the disease.

Gal et al. (2019) employed supervised machine learning models to predict complete remission in pediatric patients with AML. They used data extracted from RNA sequencing and clinical information as input features for their models. The k-nearest neighbors algorithm achieved the highest performance among the ML techniques evaluated. Additionally, the authors observed notable differences in gene expression patterns between the pre- and post-treatment periods, suggesting the potential of using gene expression data as predictive markers for treatment response in AML patients.

In a subsequent study, Mosquera Orgueira et al. (2021) employed clinical and genetic data to train a random forest classifier to predict the survival probability of AML patients. The researchers identified patient age and gene expressions of *KDM5B* and *LAPTM4B* as the three most influential variables. These findings suggest that combining ML techniques with clinical and molecular data holds significant predictive potential for AML diagnosis and supporting therapeutic decision-making. The study emphasizes the importance of incorporating genetic information into predictive models, as it provides valuable insights into the prognosis and treatment response. Such ML-based approaches offer a promising avenue for enhancing patient management and personalized treatment strategies.

Gerstung et al. (2017) presented a statistical decision support model to predict personalized treatment outcomes for AML patients. The model employs prognostic data available in a knowledge bank and demonstrates the significant impact of clinical and demographic factors, including age and blood cell count, on early death rates, particularly mortality related to treatment. Through the knowledge bank-based model, the authors observed that approximately one-third of the analyzed patients would modify their treatment protocols when comparing the model's recommendations to those of the ELN. This highlights the potential of leveraging comprehensive prognostic data and statistical modeling to enhance treatment decisions and potentially improve patient outcomes. The study underscores the importance of incorporating personalized and data-driven approaches in the management of AML.

In a comprehensive study, Itzykson et al. (2021) proposed a rule-based decision support system that integrates statistical and machine learning techniques to facilitate treatment decision-making for elderly patients diagnosed with AML. The model employs overall survival predictions based on the Kaplan-Meier method and incorporates seven oncogenetic markers (*NPM1, FLT3-ITD, DNMT3A, NRAS, ASXL1, KRAS*, and *TP53*) to stratify patients into distinct treatment groups. These groups provide insights into the intensity of treatment required and offer guidance on whether treatment decisions should be strictly adhered to, cautiously analyzed, or entirely discarded. The authors found that their model exhibited more discriminative ability than the 2017 ELN stratification, successfully identifying 30–35% of patients with superior outcomes and accurately censoring the need for hematopoietic stem-cell transplantation in the first remission. Moreover, multivariate logistic regression analysis identified mutations in the *NRAS, SETBP1, RUNX1*, and *ASXL1* genes as independent predictors of poor complete remission rates in non-adverse risk patients. These findings further emphasize the significance of incorporating decision-support tools that consider clinical and genetic data for accurate treatment prediction.

An interpretable model for predicting the survival of AML patients was presented by Almeida et al. (2023). This model leveraged the Explicable Boosting Machines (EBM) technique, and the results emphasized the importance of using genetic data in AML analysis, particularly gene expression data. Furthermore, they highlighted the importance of selecting specific treatment groups for patient survival.

Several recent studies on AML-related diseases have also demonstrated the effectiveness of applying state-of-the-art ML techniques in pattern recognition, risk prediction, and survival prediction. These diseases include acute lymphoblastic leukemia (Fitter et al., 2021), myelodysplastic syndrome (Radhachandran et al., 2021), breast cancer (Kate and Nadig, 2017), prostate cancer (Zolbanin et al., 2015; Rabaan et al., 2022), rectal cancer (Wang et al., 2022), skin cancer (Ahmed et al., 2022), nasopharynx cancer (Jing et al., 2020), pancreatic cancer (Walczak and Velanovich, 2018; Muhammad et al., 2019; Wang et al., 2020), infective endocarditis (Ris et al., 2019), AML in pediatric patients (Hoch et al., 2021), and AML with myelodysplasia-related changes (Yu et al., 2021). The success observed indicates that contemporary ML techniques can automatically uncover meaningful patterns within vast datasets.

In this context, this study presents a decision support system designed to recommend suitable therapeutic protocols automatically for AML patients based on their survival prediction. The primary aim is to mitigate the subjectivity inherent in treatment decisions and reduce the time involved in the decision-making process. Consequently, we can deliver more accurate and reliable treatment recommendations that minimize adverse effects. Our ultimate goal is to improve patient outcomes by extending their survival time and enhancing their overall quality of life.

## 3 Materials and methods

The decision support system proposed in this work combines supervised models computed by three established machine learning methods commonly employed in the medical field: Random Forests (RF), Support Vector Machines (SVM), and Logistic Regression (LR). This system automatically recommends the best treatments for AML patients based on the automatic prediction of clinical outcome (survival/decease).

**Figure 1:**
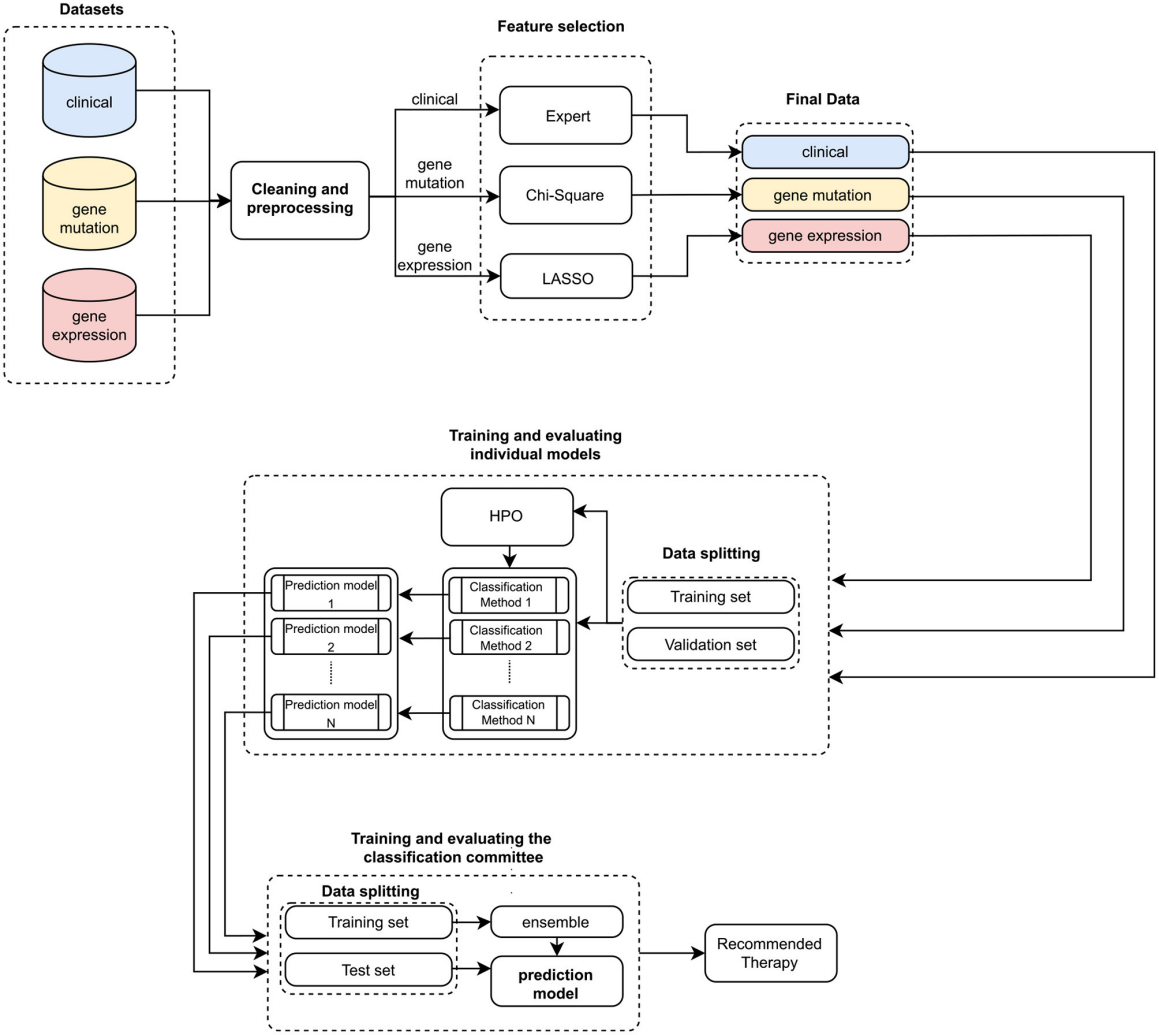
Pipeline for training and evaluating the ensemble. Initially, three sets of clinical and genetic data are input. Then, these data are preprocessed and cleaned to facilitate the feature selection process. This process is expert-guided for clinical data (Expert) and automated for genetic data (Chi-Square and LASSO). With these validated datasets, we trained individual models (HPO stands for Hyperparameter optimization). The best individual predictors are selected to compose the classification committee. Subsequently, a new training and evaluation process is carried out, now based on the classification committee (ensemble). In the end, a therapy recommendation is computed.

**Figure 2:**
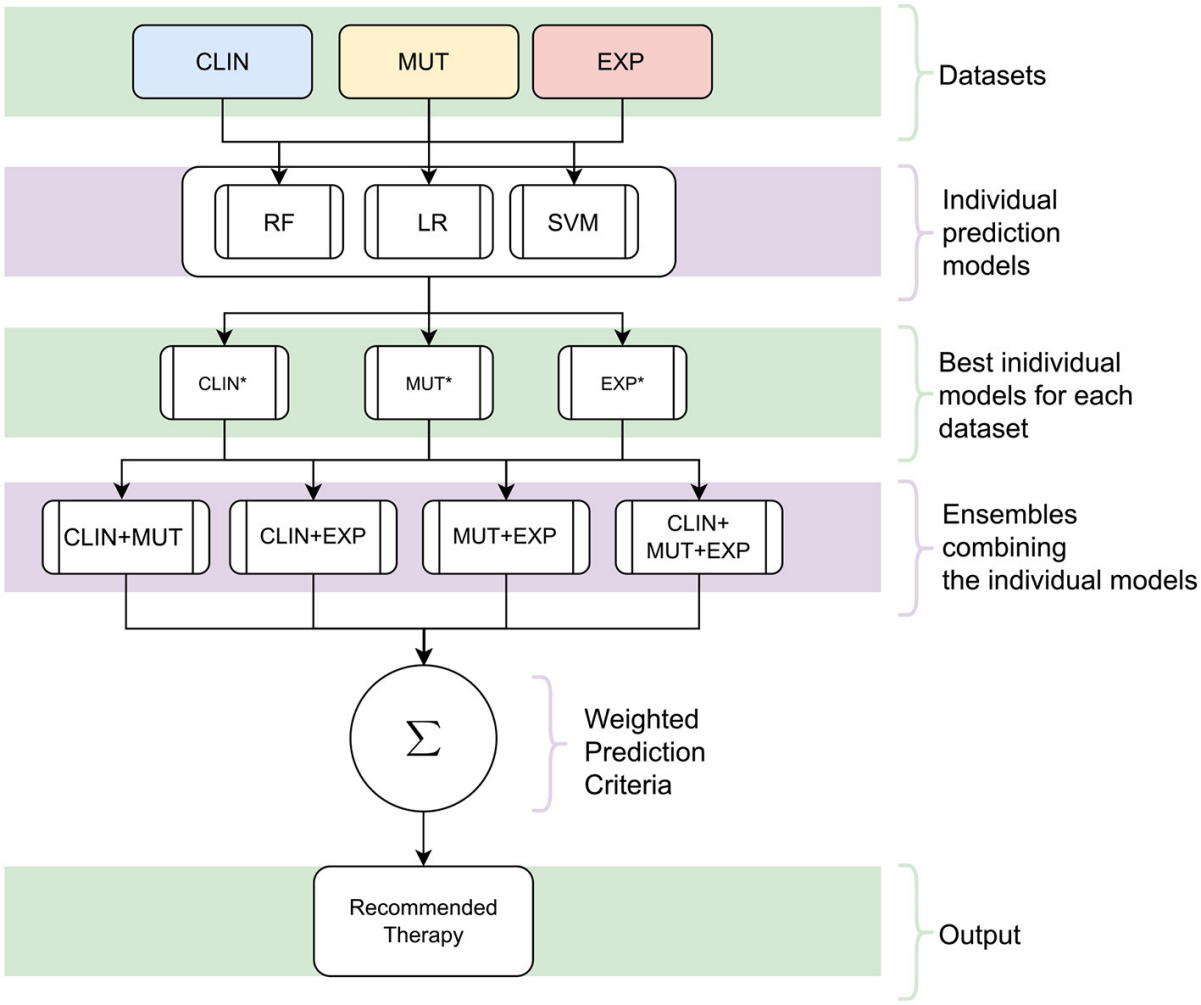
The architecture of the proposed decision support system. Initially, individual models are trained using the datasets [clinical (CLIN), gene mutation (MUT), and gene expression (EXP)] with the three Machine Learning techniques [Random Forests (RF), Logistic Regression (LR), and Support Vector Machine (SVM)]. Subsequently, the best individual models are selected for each kind of data from all those produced (the asterisk symbol represents these models). In the following, a combination of these models is created by a classification committee with the final prediction vote weighted. Since the result of this committee and the models is a survival response, the process is repeated for all possible *Treatment Intensity*. In the end, the recommended therapy is the one that maximizes the probability of patient survival.

Altogether, we have trained nine clinical outcome prediction models using selected attributes from real and public databases composed of (*i*) clinical data (CLIN), (*ii*) mutation data (MUT), and (*iii*) gene expression data (EXP). Then, we combined the best-trained models, one for each combination of the three databases. The ensemble outputs are aggregated to compose a robust final prediction model. Figure 1 summarizes the processes for generating the proposed system, and Figure 2 illustrates the architecture of the resulting ensemble models. In the following, we detail each process involved in designing the method proposed in this work.

The final output encompasses recommending a therapeutic course from distinct treatment groups defined by experts in the field and outlined below. As both the individual models and the committee yield survival predictions, the recommendation rests on selecting the group that optimizes the survival forecast for the AML patient.

### 3.1 Datasets

The datasets used to train and evaluate the prediction models come from studies by *The Cancer Genome Atlas Program* (TCGA) and *Oregon Health and Science University* (OHSU). These datasets are known as *Acute Myeloid Leukemia* (The Cancer Genome Atlas Research Network, 2013; Tyner et al., 2018) and comprise clinical and genetic data of AML patients. Both are real and available in the public domain at: https://www.cbioportal.org/. We used three sets with data collected from the same patients: one with clinical information, another with gene mutation data, and another with gene expression data. Table 1 summarizes the original data in the three feature sets extracted from the two databases.

**Table 1:** Amount of original data in each database.

			**Features**		
**Database**	**#Samples**	**#Patients**	**Clinical**	**Mutation**	**Expression**
TCGA	200	200	31	25,000	25,000
OSHU	672	562	97	606	22,825

Specialists in the data domain analyzed and grouped the treatments in the clinical data into four categories according to the intensity of each therapy (Almeida et al., 2023):

1. *Target therapy*—therapy that uses a therapeutic target to inhibit some mutation/AML-related gene or protein;
2. *Regular therapy*—therapy with any classical chemotherapy;
3. *Low-intensity therapy*—non-targeted palliative therapy, generally recommended for elderly patients; and
4. *High-intensity therapy*—chemotherapy followed by autologous or allogenic hematopoietic stem cell transplantation.

Likewise, cytogenetic information was normalized and grouped by specialists in the data domain. In addition, to reduce the original fragmentation in the data related to the patient's race, we binarized the values with 1 indicating that the patient is white and 0 otherwise. This is because white patients represent about 75% of the data.

### 3.2 Data cleaning and preprocessing

Since the data comes from two sources, we have processed them to ensure consistency and integrity. With the support of specialists in the application domain, we removed the following data:

1. Samples not considered AML in adults observed by (*i*) the age of the patient, which must not be < 18 years, and (*ii*) the percentage of blasts in the bone marrow, which should be ≥20%;
2. Samples without information on survival elapsed time after starting treatment (*Overall Status Survival*);
3. Duplicated samples. We have removed all instances from the OHSU database in which the attribute value of *Site of Sample* differed from *Bone Marrow Aspirate*. As the original dataset contains multiple samples from the same patient, all blood samples collected outside the bone marrow were removed.
4. All instances from the OHSU database were excluded where the value of the attribute *Sample Timepoint* differed from *de novo*. This is because the TCGA database contains only blood samples from patients with *AML de novo*;
5. Attributes identifying the type of cancer, as all patients were diagnosed with AML; and
6. Any other feature that is not present in both databases.

We used the 3-Nearest Neighbor method (KNN; Cover and Hart, 1967) to fill empty values in clinical data features (CLIN) automatically. We used the features with empty values as the target attributes and filled them using the value predicted from the model trained with other attributes (i.e., without empty values). After the preprocessing stage, the gene expression (EXP) and mutation (MUT) data do not have empty values. Nevertheless, we removed the features of 37 genes with no mutations.

Subsequently, we kept only the samples in which all the variables are compatible, observing data related to the exams and treatment received by the patients, as these affect the nature of the clinical, mutation, and gene expression data. Of the 872 initial samples in the two databases, 272 were kept at the end of the preprocessing, integration, and data-cleaning processes. Finally, specialists in the data domain checked and validated all the data.

### 3.3 Feature selection

This section describes the feature selection process used to represent clinical, gene mutation, and gene expression data.

#### 3.3.1 Clinical data

**Figure 3:**
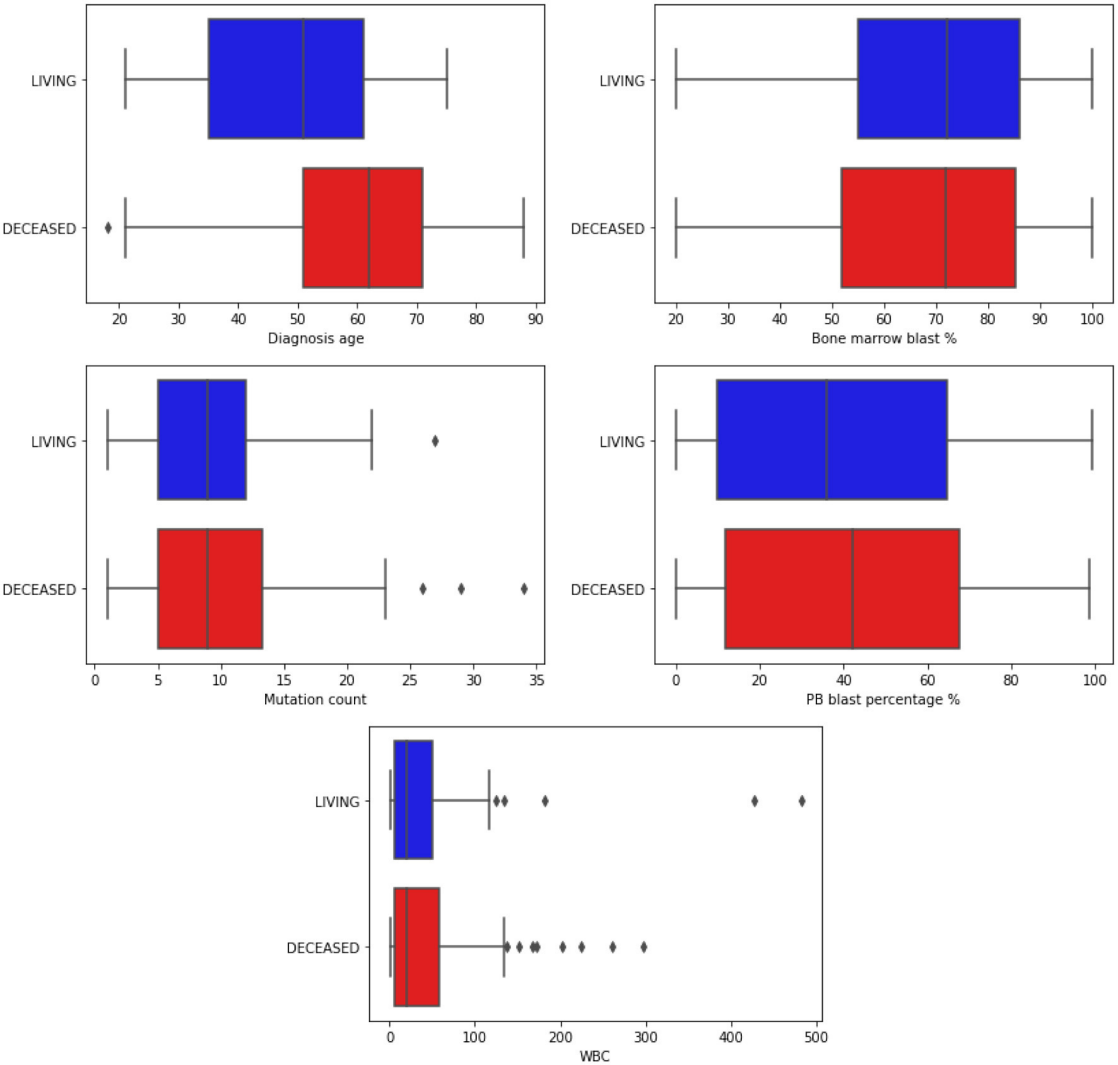
Boxplots illustrating the distribution of continuous clinical features. The data is segmented based on the classes of the target attribute. Blue bars represent the *Living* patients, while red represents the *Deceased* ones. “WBC” refers to White Blood Count, a numerical measurement of the total count of white blood cells in a given blood volume. Additionally, “PB” stands for Peripheral Blood, indicating blood collected from the peripheral circulatory system rather than from specific organs or tissues.

**Figure 4:**
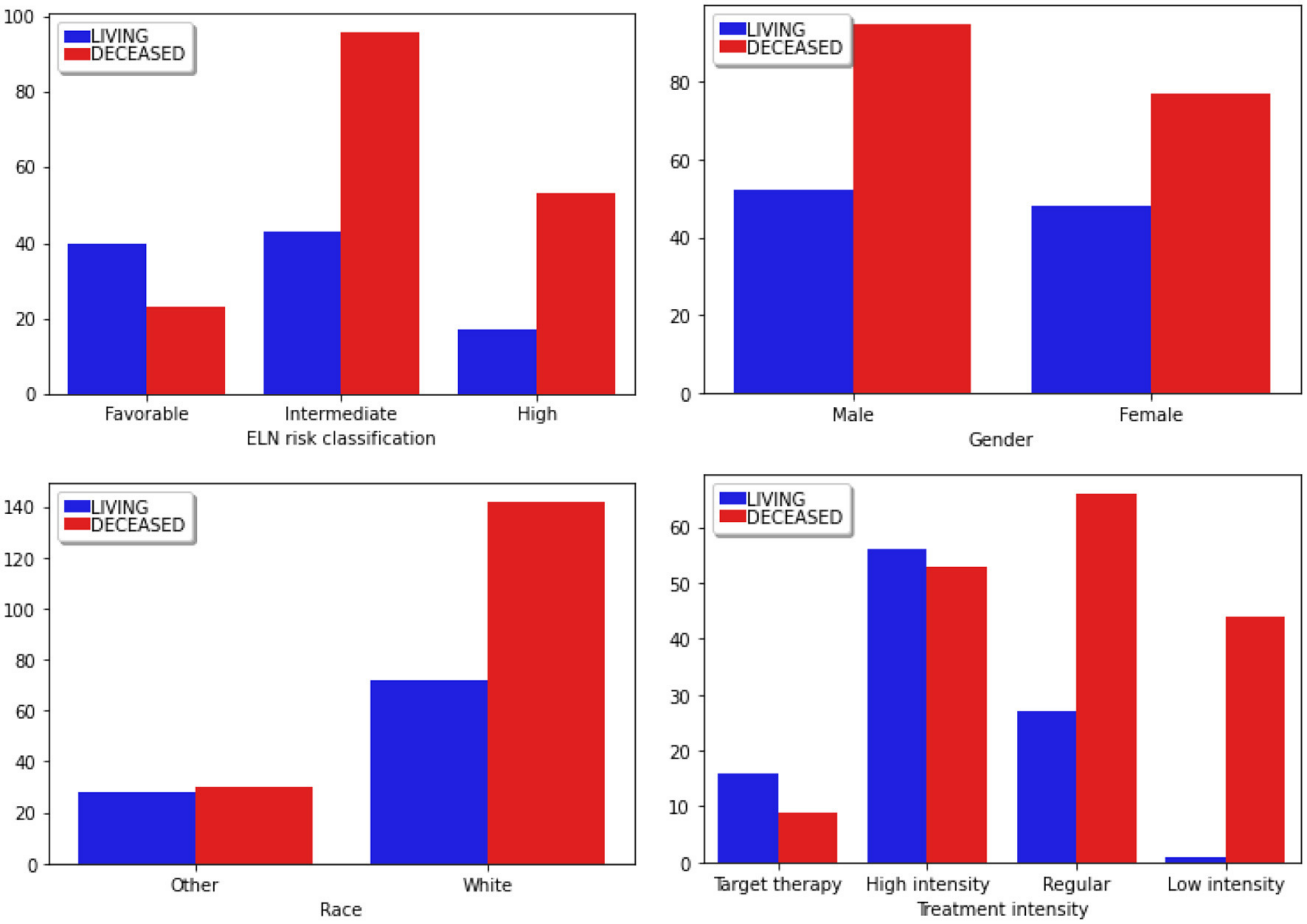
Distribution of categorical clinical features segmented according to the classes of the target attribute. Blue bars correspond to the *Living* patients, while red represents the *Deceased* ones. “ELN risk classification” corresponds to the European LeukemiaNet risk classification system, a recognized panel extensively referenced in leukemias.

Among the clinical attributes common in the two databases (TCGA and OSHU), specialists in the data domain selected the following according to their relevance for predicting clinical outcomes. In Table 2, we briefly describe all selected clinical features, and Table 3 summarizes the main statistics of those with a continuous nature. Figures 3, 4 summarize their main statistics.

**Table 2:** Clinical features description.

**Feature**	**Description**
Diagnosis age	Patient age when diagnosed with AML
Bone marrow blast %	Percentage of blasts in the bone marrow
Mutation count	Number of genetic mutations observed
PB blast %	Percentage of blasts in peripheral blood
WBC	White blood cell count
Gender	Patient gender
Race	Whether the patient is white or not
Cytogenetic info	Cytogenetic information that the specialist used in diagnosing the patient
ELN risk classification	ELN risk groups (favorable, intermediate, and adverse)
Treatment intensity classification	The intensity of treatment received by the patient (target, regular, low-intensity, or high-intensity therapy)
Overall survival status	Patient survival status (living or deceased).

**Table 3:** Main statistics of clinical features with a continuous nature.

**Feature**	**Minimum**	**Maximum**	**Median**	**Mean**
Diagnosis age	18	88	58	55.38
Bone marrow blast %	20	100	72	68.18
Mutation count	1	34	9	9.72
PB blast %	0	99.20	38.15	40.54
WBC	0.4	483	39.44	18.04

The *Diagnosis age* is concentrated in the range of 50–70 years, with an outlier below 20 years and above 18 among those who did not survive during the analyzed period. In contrast, the age range among surviving patients is between 32 and 60. It is in line with the literature that the diagnosis age can influence the course of the disease (Gal et al., 2019; Mosquera Orgueira et al., 2021).

The percentage of blasts in peripherical blood (*PB blast %*) and bone marrow (*Bone marrow blast %*) shows a similar distribution concerning the “living” and “deceased” patients. The white blood cell count (*WBC*) revealed outliers, with its distribution concentrated between 0 and 100. Finally, most patients' mutations range (*mutation count*) from 5 to 15, with outliers exceeding 25.

Most patients fall into the “Intermediate” group. Given that this classification commonly influences therapeutic decisions, a higher proportion of patients in this group experienced adverse outcomes (Döhner et al., 2017).

Regarding *Gender*, there are more males than females in these studies. Furthermore, the mortality incidence is notably higher among males, a factor that can be considered in the therapeutic decision-making process. Regarding *Race*, there is a predominant incidence of patients identified as white, while other races are grouped under the *Other* category.

Finally, the data predominantly focused on the *High Intensity* and *Regular Therapy* groups, treatments that involve oral chemotherapy, and, in the case of high intensity, there is consolidation with bone marrow transplantation. Most patients who received *Low-Intensity* treatment succumbed to the disease. This type of therapeutic choice is commonly employed for patients in the terminal stage.

#### 3.3.2 Gene expression data

After data preprocessing and cleaning, 14,712 gene expression attributes remained. To select the most relevant ones for survival prediction, we employed the LASSO method (SVM with L1 regularization). This calculates coefficients for each attribute based on its relevance for classification.

We have trained the method using all gene expression attributes with a regularization factor of *C* = 0.01. At the end of the training process, 22 expression attributes were selected: *CCDC144A, CPNE8, CYP2E1, CYTL1, HAS1, KIAA0141, KIAA1549, LAMA2, LTK, MICALL2, MX1, PPM1H, PTH2R, PTP4A3, RAD21, RGS9BP, SLC29A2, TMED4, TNFSF11, TNK1, TSKS*, and *XIST*.

#### 3.3.3 Gene mutation data

After cleaning and preprocessing the data, 281 gene mutation features remained. Then, we employed the χ^2^ statistical method to select a subset of these features. For this, we defined the following hypotheses: H0—patient survival is independent of gene mutation; H1—both groups are dependent. Using *p* < 0.1, a set of 10 gene mutation features were selected: SRSF2, U2AF1, RIF1, PRKAA2, CALR, CADM2, PTPN11, PHF6, CTNNA2, and TP53.

After the cleaning and preprocessing stage, we obtained the final database used to train and evaluate the prediction models. It has 272 samples (patient data) consisting of 11 clinical features (CLIN dataset), 22 gene expression features (EXP dataset), and 10 gene mutation features (MUT dataset). Table 4 summarizes each of these datasets. All the code used in this paper and the final database are publicly available at: https://github.com/jdmanzur/ml4aml.

**Table 4:** Final datasets used to train and evaluate the outcome prediction models.

**Dataset**	**#Features**	**Features**
Clinical (CLIN)	11	Diagnosis age, Bone marrow blast (%), Mutation count, PB blast (%), WBC, Gender, isWhite, Cytogenetic info, ELN risk classification, Treatment intensity classification, and Overall survival status (class)
Gene expression (EXP)	22	*CCDC144A, CPNE8, CYP2E1, CYTL1, HAS1, KIAA0141, KIAA1549, LAMA2, LTK, MICALL2, MX1, PPM1H, PTH2R, PTP4A3, RAD21, RGS9BP, SLC29A2, TMED4, TNFSF11, TNK1, TSKS, and XIST*
Gene mutation (MUT)	10	*SRSF2, U2AF1, RIF1, PRKAA2, CALR, CADM2, PTPN11, CTNNA2, PHF6, and TP53*

#### 3.3.4 Expression impact survival analysis

**Figure 5:**
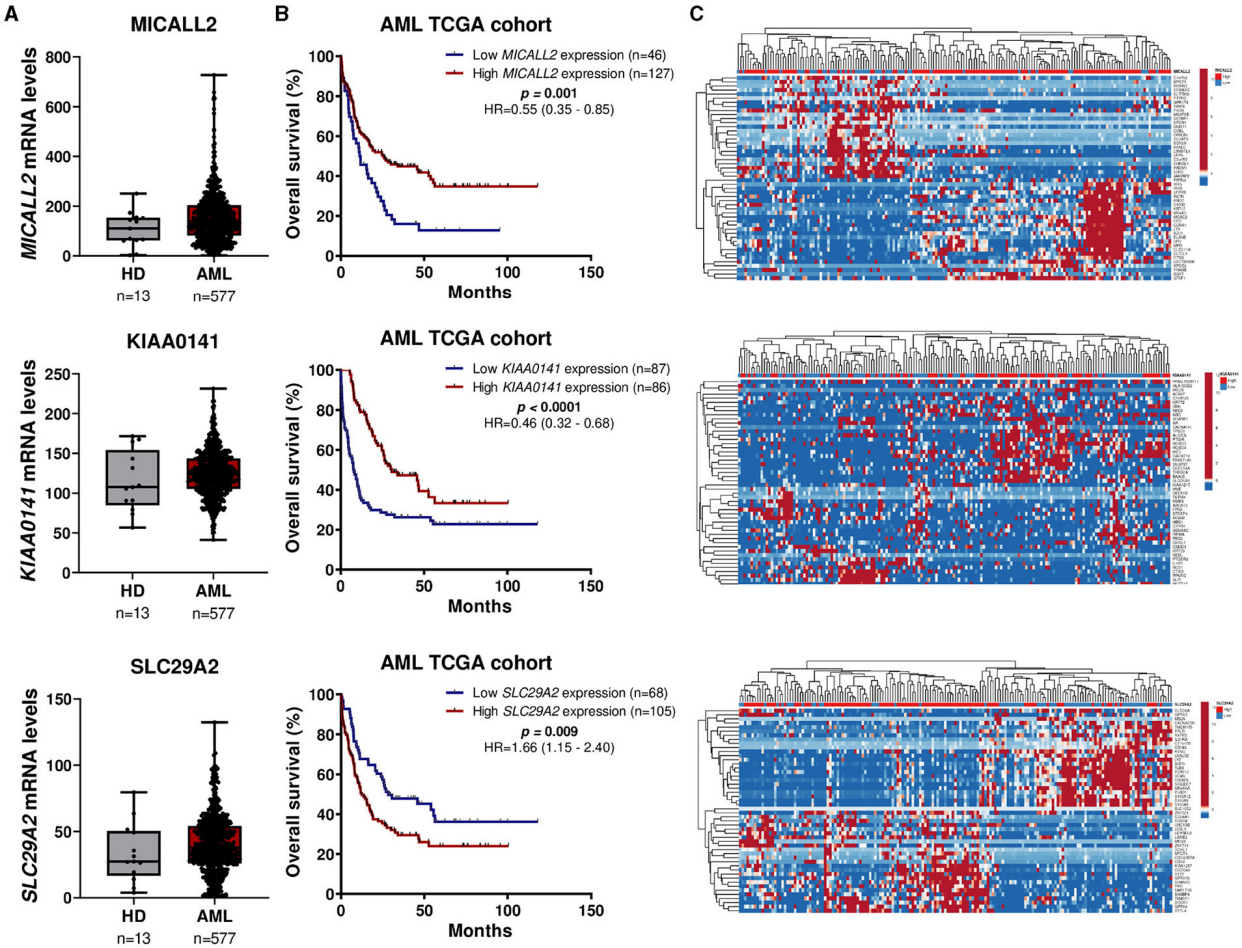
*MICALL2, KIAA0141*, and *SLC29A2* expression impact survival outcomes and biological characteristics in AML patients. **(A)** *MICALL2* (probe 219332_at), *KIAA0141* (201977_s_at), or *SLC29A2* (probe 204717_s_at) mRNA levels were compared between AML patients (*n* = 577), and samples of normal hematopoietic cells (normal bone marrow *n* = 5, CD34+ cells *n* = 8). The “*y*” axis represents mRNA expression levels at arbitrary values. Horizontal lines represent the median. **(B)** Kaplan-Meier curves represent overall survival for AML patients dichotomized according to high or low *MICALL2, KIAA0141*, or *SLC29A2* expression (using the ROC curve as the cut-off point). Hazard ratio (HR), 95% confidence interval, and *p* values are indicated (log-rank test). **(C)** Heatmap constructed using ClusterVis that summarizes the expression of the top-25 upregulated and 25 downregulated genes for high vs. low *MICALL2, KIAA0141*, or *SLC29A2* expression. Color intensity represents the *z*-score within each row.

**Figure 6:**
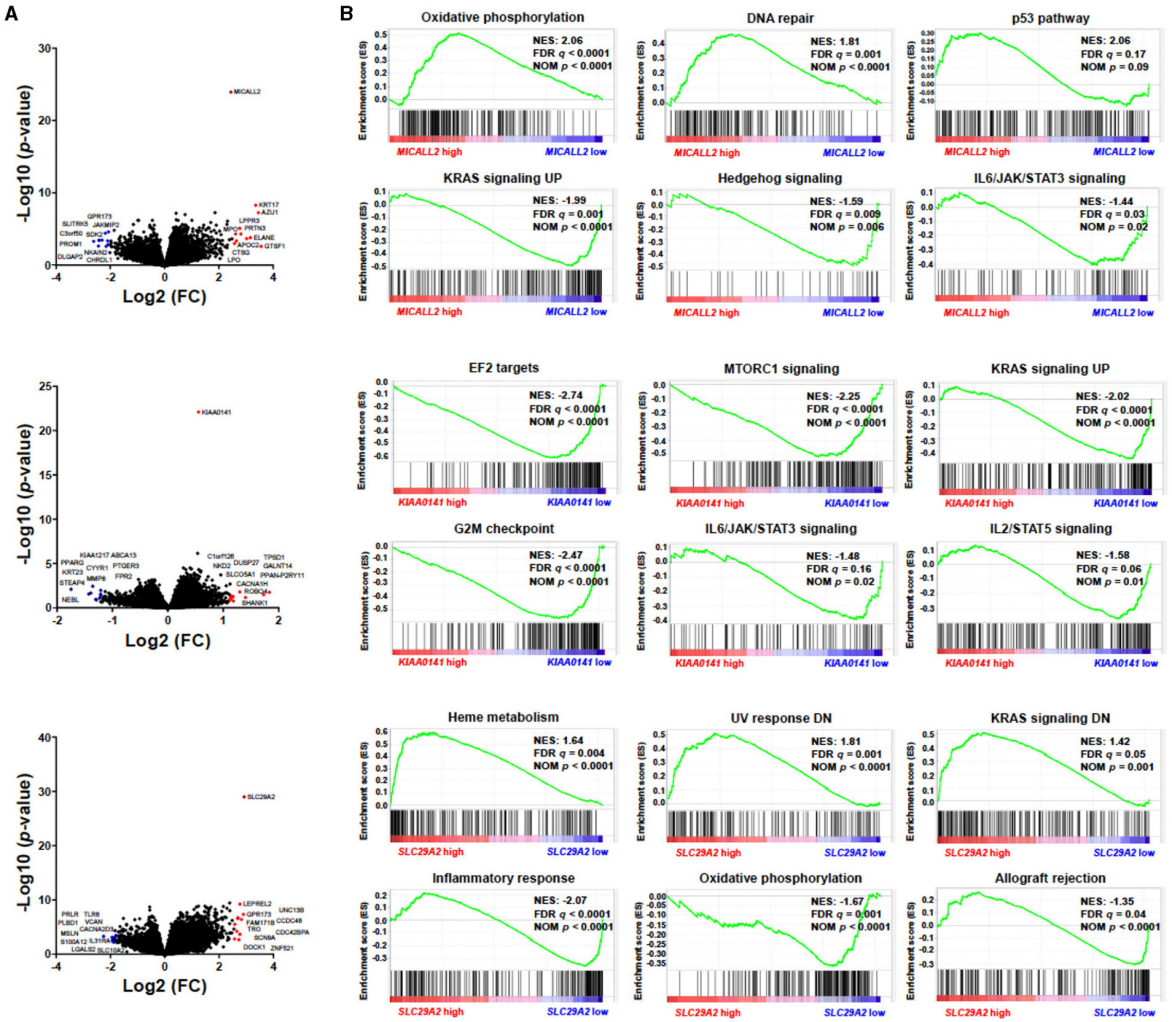
*MICALL2, KIAA0141*, and *SLC29A2* expression impact survival outcomes and biological characteristics in Acute Myeloid Leukemia (AML) patients. **(A)** Volcano plots depicting the extent (*x*-axis) and significance (*y*-axis) of differential gene expression for each gene, comparing high vs. low *MICALL2, KIAA0141*, or *SLC29A*. **(B)** Gene Set Enrichment Analysis plots for biological processes associated with *MICALL2, KIAA0141*, or *SLC29A2* expression in AML patients. The top portion of the plot shows the running enrichment scores (ES) for the gene set. The point with the maximum deviation from zero is defined as the ES for the gene set. The leading-edge subset (the subset of genes with the most significant contribution to the ES) is shown as a vertical bar accumulating before the peak score for a positive ES or after the peak score for a negative ES. FDR-adjusted *p*–values (NOM *p*-value) and enrichment scores normalized for gene set size (NES) are indicated.

Three genes caught our attention in the gene expression selection process: *MICALL2, KIAA0141*, and *SLC29A2*. Thus, we deeply analyzed the impact on survival outcomes and biological characteristics of patients with AML. First, we compare their mRNA levels between AML patients and samples of normal hematopoietic cells. Then, we plot the Kaplan-Meier curves to check the overall survival for AML patients dichotomized according to high or low expression. Next, we compute a heatmap using ClusterVis to summarize the expression of the top-25 upregulated and 25 downregulated genes for high vs. low expression (Figure 5). Additionally, we use Volcano plots to depict the extent and significance of differential gene expression for each gene, comparing high vs. low. Finally, we also compute Gene Set Enrichment Analysis plots for biological processes associated with the three gene expressions in AML patients (Figure 6).

### 3.4 Training the outcome prediction models

We have used three established supervised machine learning methods to train models that automatically predict the clinical outcome (survival/decease) based on the chosen treatment intensity (target, regular, low-intensity, and high-intensity) for a given patient. The methods are Random Forest (RF), Logistic Regression (LR), and Support Vector Machines (SVM). Table 5 briefly describes the ML methods used in this study.

**Table 5:** Supervised machine learning methods used in this study.

**Algorithm**	**Description**
RF (Breiman, 2001)	An ensemble learning method that operates by training decision trees. For predicting a class, the output of the random forest is the class predicted by most trees.
LR (Cramer, 2003)	A statistical model that computes the probability of a sample belonging to some class having the log-odds for the class be a linear combination of one or more independent features.
SVM (Boser et al., 1992)	A supervised learning method that maps training samples to points in space, aiming to maximize the width of the margin that separates the two classes. It is versatile because different Kernel functions can be specified for the decision boundary.

Due to the small amount of data available to train and evaluate the models, we did not employ deep learning techniques, as these methods demand a huge amount of data. Furthermore, the explainability of prediction models is a desirable characteristic in this context.

We fit the main hyperparameters through a grid search (Mitchell, 1997). Table 6 presents the range of values evaluated. We kept the default values for all other parameters.

**Table 6:** Hyperparameters evaluated in a grid search.

**Method**	**Hyperparameters**
RF	*n_estimators*={10, 15, 20, 25, 30, 45, 50}, *min_samples_leaf*={1, 2, 3, 4}, *max_depth*={8, 10, *None*}, *class_weight*={balanced, none}
LR	*C*={10^−6^, 5.62 × 10^−5^, 3.166 × 10^−3^, 1.77 × 10^−1^, 10}, *class_weight*={balanced, none}, *penalty*={L2}, *random_state*=1
SVM	*kernel*={linear, rbf}, *C*={10^−6^, 10^−5^, 10^−4^, 10^−3^, 10^−2^}*class_weight*={balanced and none}, *random_state*=1

We have trained the three classification methods (RF, LR, and SVM) with the three datasets (CLIN, MUT, and EXP), resulting in a total of nine individual prediction models (3 ML Models × 3 datasets).

### 3.5 Ensemble

**Figure 7:**
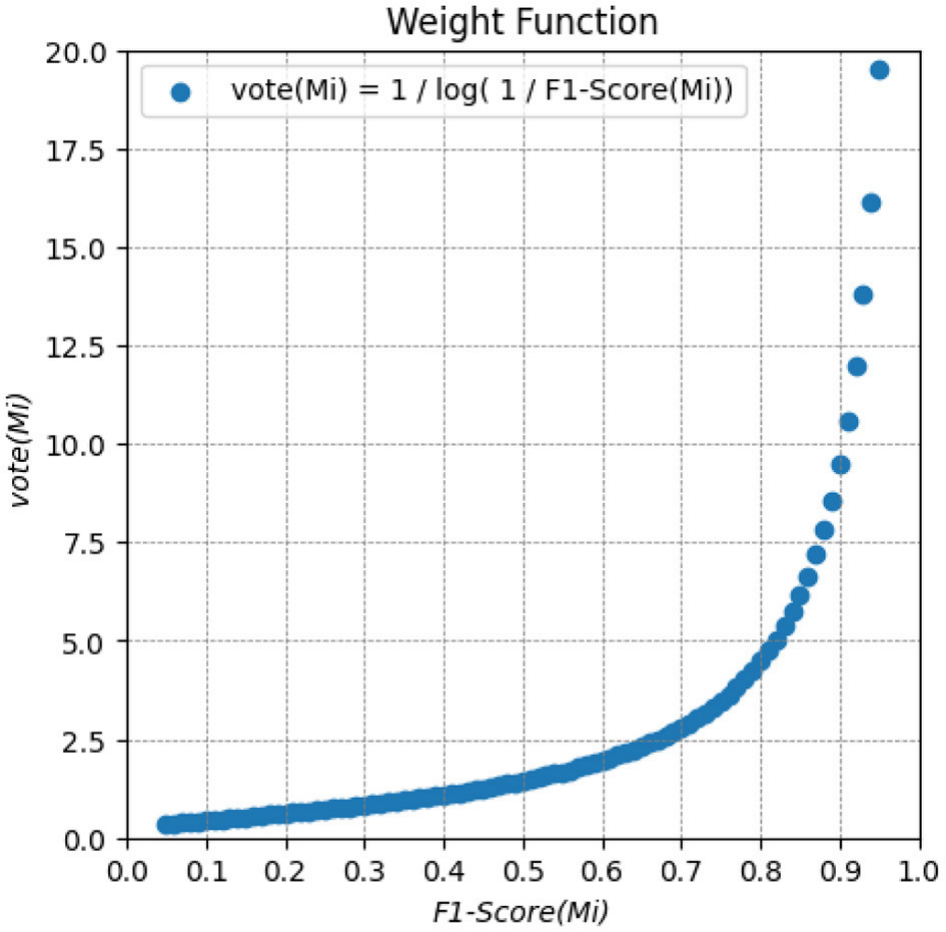
Weighted function for computing the vote of an individual prediction model *M*_*i*_. It provides a weighted vote based on the F1-Score metric for each prediction. The higher the F1-Score, the higher the weight of the models.

Among the nine outcome prediction models, we selected the ones that obtained the best results for each data set (Figure 2). We then combined these three models as a classifier committee that computes the predicted survival outcome for a given patient. Equation 1 presents how we have weighted the vote for an individual prediction model *M*_*i*_. The F1-Score corresponds to the f-measure attained by the prediction model in the validation set. The ensemble classification output is then computed from the outcome (live/decease) with the highest final vote, considering the output of all individual prediction models (Figure 7).


(1) $$\begin{array}{l} vote\left(M_{i}\right)=\left\lceil \frac{1}{\log_{10}\left(\frac{1}{F1-Score\left(M_{i}\right)}\right)}\right\rceil \end{array}$$

### 3.6 Performance evaluation

The performance of the prediction models was assessed using the traditional hold-out validation approach (Mitchell, 1997). The dataset was partitioned into four subsets: 70% was randomly selected for training the models, 10% for feature selection, another 10% for validating the individual models, and the remaining 10% as an independent test set for evaluating the performance of the ensemble. Addressing the model performance in the separated test partition simulates its application in a prospective independent patient cohort.

We have calculated the following measures to assess and compare the performance obtained by the prediction models. In the equations below, TP (true positive) is the number of patients correctly predicted by the model who deceased; FP (false positive) is the number of patients who survived, but the model incorrectly predicted to decease; TN (true negative) is the number of patients correctly predicted by the model who survived; FN (false negative) is the number of patients who deceased, but the model incorrectly predicted to survive.

**Accuracy (ACC):** the percentage of correct predictions.


$$\begin{array}{l} \text{ACC}=\frac{TP+TN}{TP+FN+TN+FP} \end{array}$$

**Recall (REC) or Sensitivity:** the proportion of true positives (patients predicted to decease) to the actual positive samples (patients who deceased) that should have been detected.


$$\begin{array}{l} \text{Recall}=\frac{TP}{TP+FN} \end{array}$$

**Precision (PREC):** the proportion of true positives (patients predicted to decease) to the actual positive results (patients who deceased), including those incorrectly identified by the prediction model.


$$\begin{array}{l} \text{Precision}=\frac{TP}{TP+FP} \end{array}$$

**F1-Score (F1):** the harmonic mean between Precision and Recall. The F1-Score is often used when the dataset is imbalanced.


$$\begin{array}{l} \text{F1}=2*\frac{Precision*Recall}{Precision+Recall} \end{array}$$

**Matthews Correlation Coefficient (MCC):** provides a balanced assessment of a model's performance, considering both positive and negative cases. The MCC ranges from -1 to +1, where +1 indicates a perfect prediction, 0 indicates a random prediction, and -1 indicates total disagreement between the model's predictions and the true labels.


$$\begin{array}{l} \text{MCC}=\frac{TP*TN-FP*FN}{\sqrt{\left(TP+FP\right)\left(TP+FN\right)\left(TN+FP\right)\left(TN+FN\right)}} \end{array}$$

**AUC:** the receiver operating characteristics (ROC) curve is used to address the success of a prediction model across several classification thresholds. The area under the ROC curve (AUC) tests the whole two-dimensional field under the entire ROC curve. AUC ranges from zero to one, and the higher, the better (Spackman, 1989).

## 4 Results and discussion

In this section, we detail and analyze all the results obtained. First, we present the main findings in selecting and analyzing clinical and genetic data. Then, we reported the results of the individual prediction models and, finally, the performance of the outcome prediction computed by the ensemble.

### 4.1 Genes that impact survival outcomes and biological characteristics

The protein encoded by *MICALL2* potentially regulates cytoskeleton dynamics, tight junction formation, and neuritis outgrowth. To the best of our knowledge, no study has analyzed the biological role of *MICALL2* in AML and other leukemias. Therefore, it could be addressed in future clinical and functional studies.

The gene *KIAA0141*, also known as *DELE1*, could indicate a patient's response to drug and radiation therapies (Jia et al., 2014; Sato et al., 2021). There are results in the literature relating this gene as a prognosis indicator because it can have a main function on mitochondrial stress (Guo et al., 2020). Sui et al. (2023) suggest that *DELE1* has an important role in improving therapy protocols for cancer.

The high expression of *SLC29A2*, also known as *ENT2*, suggests its importance in facilitating hypoxanthine transport, which is necessary for enhanced DNA synthesis through hypoxanthine recycling. In conclusion, *ENT2* shows potential as a target for developing therapeutics (Naes et al., 2023). Elevated levels of *ENT2* in the blasts at the time of diagnosis of AML were associated with a lower response to induction therapy (Rodríguez-Macías et al., 2023). Moreover, higher *ENT2* levels were linked to a poor response to treatment. These findings align with the observation that *ENT2* upregulation is associated with advanced stages of various cancer types, including mantle cell lymphoma, hepatocellular carcinoma, and colorectal cancer (Pastor-Anglada and Pérez-Torras, 2018).

Among the gene mutations identified as relevant to the model, the *TP53* mutation is the best-known. Several studies show the relationship between *TP53* mutation and therapeutic response and prognosis. The *TP53* gene is considered the guardian of genomic stability, as it controls cell cycle progression and apoptosis in situations of stress or DNA damage, and mutations in this gene are found in 1/2 of the cancer patients (Kastenhuber and Lowe, 2017; Monti et al., 2020). Although mutations in *TP53* are less common in AML patients (about 10%), they predict a poor prognosis (Papaemmanuil et al., 2016; Grob et al., 2022).

Mutations in *U2AF1* and *SRSF2* are more common in myelodysplastic syndrome and rare in *de novo* AML (Papaemmanuil et al., 2016; Xu et al., 2017), but have been associated with an unfavorable prognosis in myeloid neoplasms (Zhu et al., 2021). *U2AF1* regulates the pre-mRNA splicing processes to generate functional mRNAs, and is considered a key element in the spliceosome (Zhao et al., 2022).

### 4.2 Prediction models

We have evaluated the nine single outcome prediction models (each one trained with a different dataset and classification method), applying the traditional 8:1:1 hold-out validation strategy (Section 3.4). Specifically, the training set consisted of 216 samples randomly selected, the validation set was composed of 28 samples, and the test set also contained 28 samples. It is noteworthy that the data partitioning for training and testing was kept consistent across all models.

Table 7 summarizes the performance achieved by each model. Three stand out as the top performers, each associated with a distinct dataset (lines highlighted in bold). Notably, all these models exhibit good results, but the one using genetic expression data shows particularly promising performance.

**Table 7:** Performance achieved by individual models using the three datasets individually.

**Dataset**	**Methods**	**F1**	**AUC**	**ACC**	**PREC**	**REC**	**MCC**
CLIN	RF	0.6562	0.6222	0.6666	0.6554	0.6666	0.2603
	SVM	0.6713	0.6888	0.6666	0.7132	0.6666	0.3670
	**LR**	**0.7044**	**0.6777**	**0.7083**	**0.7031**	**0.7083**	**0.3651**
MUT	**RF**	**0.7129**	**0.7222**	**0.7083**	**0.7395**	**0.7083**	**0.4303**
	SVM	0.4807	0.5000	0.6250	0.7656	0.6250	0.0000
	**LR**	**0.7129**	**0.7222**	**0.7083**	**0.7395**	**0.7083**	**0.4303**
EXP	**RF**	**0.7803**	**0.7444**	**0.7916**	**0.7986**	**0.7916**	**0.5465**
	SVM	0.6284	0.6111	0.6250	0.6339	0.6250	0.2182
	LR	0.6200	0.5888	0.6250	0.6171	0.6250	0.1825

The best results are highlighted in bold.

The logistic regression model trained with clinical data achieved a reasonable performance. It can be valuable to healthcare specialists as it represents the initial data acquired during a patient's clinical visit. Consequently, when genetic data are inaccessible, a clinical outcome prediction model can assist the specialist in deciding the most appropriate treatment intensity for each patient. Nevertheless, including genetic data can substantially enhance predictive performance, as these attributes contribute to improved class separability.

The models created with genetic mutation data obtained superior performances compared to those trained with the clinical model. The logistic regression and random forest models obtained the same results. Regarding the classification methods evaluated, the Random Forest and Logistic Regression achieved the best overall performances. In addition, these methods have the advantage that their prediction models can be somehow explained.

### 4.3 Classification committee

We have combined the three highest-performing prediction models presented in Table 7 as a classifier committee to compute the predicted survival outcome for a given patient. The outcome prediction is computed by the weighted output (detailed in Section 3.4) provided by the individual classifiers, considering all possible treatment intensities (target, regular, low-intensity, and high-intensity; Figure 2).

We have evaluated the performance of the committees using a 9:1 hold-out (incorporating the prior validation set into the training set). Table 8 presents the results of all ensembles created by combining the individual prediction models.

**Table 8:** Performance obtained by the ensemble of classifiers.

**Dataset**	**F1**	**AUC**	**ACC**	**PREC**	**REC**	**MCC**
CLIN+MUT	0.6946	0.6429	0.6786	0.7250	0.6786	0.2582
CLIN+EXP	0.8179	0.8128	0.8214	0.8345	0.8214	0.6512
MUT+EXP	0.8179	0.8128	0.8214	0.8345	0.8214	0.6512
CLIN+MUT+ EXP	**0.8907**	**0.8846**	**0.8929**	**0.9107**	**0.8929**	**0.8006**

Bold values represent the best achieved results.

The results obtained are promising as most of the performance measures improved significantly, indicating we can safely use it as a decision support system to recommend appropriate therapy protocol for AML patients, with precision higher than 90%. We obtained the best overall results by combining the models trained with the three datasets available.

Regarding clinical data, the ensemble that combined these data with genetic expression presented a significant improvement compared to individual models trained in both contexts. These results suggest that combining these data types leads to substantial predictive power for survival in AML patients. In the case of combining genetic mutation and clinical data, a decrease in predictive ability was observed compared to the individual model trained only with genetic mutation data. Nevertheless, the ensemble that combined genetic data exhibited enhanced performance compared to individual models of this data type.

## 5 Conclusions

To guide the selection of therapy protocols for patients with AML, healthcare specialists commonly rely on prognostic evaluations based on treatment response predictions and clinical outcomes. The prevailing ELN risk stratification categorizes patients into favorable, intermediate, and adverse risk groups. However, this classification tends to be conservative, with most patients falling into the intermediate risk category. Consequently, specialists often demand additional examinations, leading to delays in treatment initiation and potentially compromising the patient's clinical condition.

This paper presented a decision support system that automatically recommends appropriate intensity oncology therapies based on the clinical outcome prediction for a given patient. The core of this system is composed of a committee of classifiers trained with clinical data and gene mutation and expression data. The proposed ensemble achieved a high performance close to 0.9 in F1-Score and AUC.

We also conducted an evaluation of individual models trained solely with specific types of data. Among them, the model generated with gene expression data exhibited superior performance and could independently assist healthcare specialists in determining the most suitable treatment for individual patients. For further improvement, specialists could employ the ensemble model incorporating all data types. In cases where genetic data collection is unavailable in the clinical setting, the single model trained solely with clinical data can be employed as an alternative.

The findings presented in this work indicate that we can employ state-of-the-art machine learning techniques to automatically process and analyze large volumes of clinical and gene data. These approaches can effectively support specialists in making well-informed decisions regarding the most suitable and safe therapy for individual patients. By significantly reducing the time required for treatment selection, these techniques can enhance overall patient outcomes, leading to extended survival and improved quality of life for individuals afflicted with the disease.

Despite the promising results presented in this study, it is essential to highlight its main limitations. The amount of public data available and used is restrictive to train more sophisticated and accurate machine-learning models. Furthermore, the data represents the characteristics of a particular regionality. At least 75% of the blood samples are from patients of white race, which may hinder the generalization power of the decision support system across different races.

In future work, we aim to assess the performance of the proposed system in a real-world scenario. Furthermore, we recommend further investigating the genes selected in the feature selection stage. This analysis would provide valuable insights into the biological significance and functional implications of the selected genes, mainly *MICALL2, KIAA0141*, and *SLC29A2*, potentially revealing novel biomarkers or therapeutic targets related to AML. Such endeavors would contribute to refining and validating the proposed system, ultimately enhancing its application and impact on clinical decision-making processes.

## Data availability statement

The datasets presented in this study can be found in online repositories. The names of the repository/repositories and accession number(s) can be found in the article/supplementary material.

## Author contributions

GC: Conceptualization, Data curation, Formal analysis, Investigation, Methodology, Resources, Software, Validation, Visualization, Writing—original draft, Writing—review & editing. JA: Conceptualization, Data curation, Formal analysis, Investigation, Methodology, Resources, Software, Validation, Visualization, Writing—original draft, Writing—review & editing. JM-N: Conceptualization, Data curation, Formal analysis, Funding acquisition, Validation, Writing—original draft, Writing—review & editing. TA: Conceptualization, Data curation, Formal analysis, Funding acquisition, Investigation, Methodology, Project administration, Supervision, Validation, Writing—original draft, Writing—review & editing.
